# Assessing dengue vector breeding in diverse environments for targeted interventions: Case studies from Colombia and Nepal

**DOI:** 10.1016/j.crpvbd.2026.100449

**Published:** 2026-10-15

**Authors:** Emerson Barahona, Dorinda Nelson-Cofie, Axel Kroeger, Kathleen Chavarría, Niseth Vence, Megha Raj Banjara

**Affiliations:** a Centre of Planetary Health, University of Freiburg, Freiburg im Breisgau, 79098, Germany; b Universidad Popular del Cesar, Valledupar, Colombia; c Central Department of Microbiology, Tribhuvan University, Kirtipur, Kathmandu, Nepal

**Keywords:** Dengue, *Aedes* vectors, Pupal productivity, Water storage containers, Vector control

## Abstract

Dengue and other *Aedes*-borne diseases are increasing globally, with vector control being the primary prevention strategy in the absence of an effective vaccine. Because mosquitoes breed in domestic water containers, identifying the most productive container types is essential for targeted interventions. This study compared pupal productivity of *Aedes* spp. and productive container types in two settings in Colombia and Nepal with distinct environmental, epidemiological, and water storage characteristics. A pupal/demographic survey was conducted in Valledupar, Colombia, and Triyuga Municipality, Nepal, including 300 and 121 households, respectively. Pupal counts were used as a proxy for adult mosquito production. In Colombia, 16 of 506 containers were productive, with large water storage containers accounting for over 90% of pupae. In Nepal, 34 of 396 containers were productive, with containers such as barrels and flower vases contributing most pupae. In both locations, pupal production was highly clustered in a small number of containers. These findings show that the most productive breeding sites differ between places, with large containers dominating in Colombia and smaller containers in Nepal. Targeted vector control strategies should therefore, focus on the specific container types responsible for most pupal production in each context. Context-specific interventions guided by pupal/demographic surveys are essential for efficient dengue control.

## Highlights

• Container types supporting *Aedes* spp. production differed between Colombia and Nepal.
• Breeding sites supporting *Aedes* spp. production reflected local water storage practices.
• Pupal production was highly clustered in a few water containers.
• Pupal/demographic surveys may guide context-specific dengue vector control.

## Graphical abstract

**Figure:**
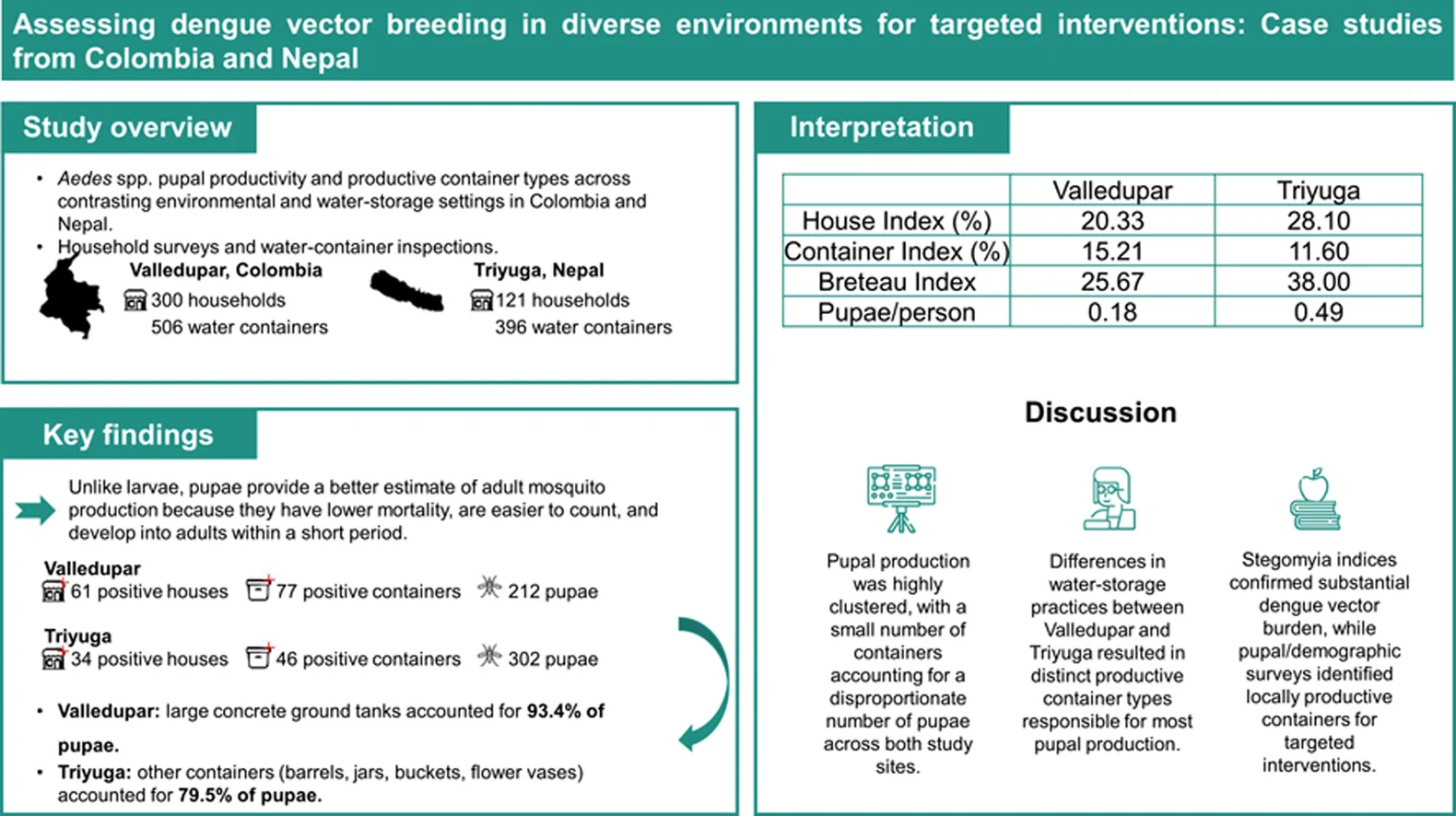

## 1 Introduction

In recent decades, the cases of arboviral diseases, particularly *Aedes-*borne diseases have increased globally. *Aedes aegypti* and *Aedes albopictus* mosquitoes serve as primary vectors of dengue, Zika, and chikungunya. According to the World Health Organization, the number of global dengue cases rose from 500,000 in 2000 to 5.2 million in 2019, spreading across 128 countries (WHO, 2024). Although there was a brief decrease during the COVID-19 pandemic (2020–2022), dengue outbreaks surged again in 2023, even spreading to previously unaffected regions. Without a viable public health vaccine, vector control continues to be the most important strategy for prevention (Morrison et al., 2004; Garjito et al., 2020).

*Aedes* spp. mosquitoes need water for oviposition (laying eggs), and the breeding sites are predominantly water-holding containers in and around houses. The characteristics of containers, such as location, type, and size, play crucial roles in mosquito breeding patterns (Ngugi et al., 2020). The presence of shrubbery above containers, a lack of use for one week or more (corresponding to the time of larval development), and the absence of container covers increase vector breeding. Access to human blood, which is needed for mosquito ovarian development, is facilitated by people living close to each other, suggesting the prioritization of vector control in neighborhoods with high population density (Arunachalam et al., 2010). Stegomyia indices have been widely used as surveillance tools in dengue prevention and control programmes in almost every country. These indices are influenced by the geographical and climatic conditions of an area and can help identify areas at high risk of dengue and guide the implementation of vector control programmes (Adak and Jana, 2020).

Containers productive for mosquito breeding can be identified by counting the pupae in water containers, making the pupal index a more accurate method for estimating container productivity compared to larval infestation (Manrique-Saide et al., 2011). Pupal counts are closely correlated with adult mosquito populations, as pupae have a lower mortality rate than larvae, are easier to count, and the development period from pupae to adults is short (Focks and Chadee, 1997; Arredondo-Jiménez and Valdez-Delgado, 2006; Focks and Alexander, 2006).

Pupal indices have shown that the percentage of collected pupae increases in large containers at the beginning of the dry season, when piped water is often rationed in low-income areas, prompting residents to store water in drums and tanks. During the rainy season, most pupae are typically collected from containers of various sizes, including buckets, tires, drums, and tanks (Lenhart et al., 2006). In many settings, large drums and barrels used for year-round water storage have emerged as key breeding sites (Romero-Vivas et al., 2006; Mwakutwaa et al., 2023). In contrast, small, discarded containers are often more abundant but contribute only a limited proportion of pupae (and thus adult mosquitoes), whereas larger containers, although fewer in number, may play a significantly greater role in pupae production (Romero-Vivas et al., 2002). Vector control targeting only the most productive water container types achieves the same or greater reduction in the vector population as a non-targeted approach, while the cost of non-targeted interventions is higher and requires more effort and resources (Tun-Lin et al., 2009).

Due to the significant health burden posed by vector-borne diseases, various control efforts have focused on prevention and control interventions (Gopalan et al., 2021; WHO, 2023). One initiative took place in Mexico, evaluating the effectiveness, biosafety, and resident perceptions of new interventions involving microencapsulated insecticides in paints. This product involves a water-based polymer coating that gradually releases the active ingredient, providing sustained protection by targeting the mosquitoes during their resting periods when applied to indoor walls (Villegas-Trejo et al., 2024). Despite the extensive literature on dengue vector control and prevention strategies, the most productive container types can differ substantially between locations, highlighting the need for local evidence to guide targeted interventions. Given the rising incidence of dengue, ongoing research on effective and context-specific control measures remains essential (Carrillo et al., 2023). Before implementing *Aedes*-vector control measures, it is necessary to identify the most productive containers locally and assess the feasibility of context-appropriate interventions.

This study serves as a baseline for targeted dengue vector control interventions in diverse environmental and epidemiological settings, using Colombia and Nepal as examples of how productive breeding containers can differ between locations. Dengue has been well known in Colombia for decades but is a relatively new and emerging infectious disease in Nepal; the first case was recorded in 2004, and the disease is now endemic, with cases reported in most districts (Bijukchhe et al., 2023). Colombia is a hyperendemic country with a high burden of dengue (Rodríguez-Morales et al., 2024).

## 2 Materials and methods

### 2.1 Study sites

The study was conducted at two urban sites with different geographical and climatic conditions. In Colombia, the survey took place in the city of Valledupar (10.4°N, 73.2°W; 490,075 inhabitants), Department (“departamento”) of Cesar, located in the north of Colombia in South America, with an elevation of 168 m above sea level and a total area of 4493 km^2^. The climate is warm and humid, with a yearly mean temperature of 28.4 °C. The mean annual precipitation is 961 mm, with rainy periods from April to June and August to November, and dry periods from December to March and in July (IDEAM, 2005). The city is divided into 6 districts or “comunas” (Fig. 1), forming 175 neighborhoods (DANE, 2021). In Nepal, the study was conducted in urban areas of the Triyuga Municipality (26.7919°N, 86.6990°E; 102,725 inhabitants), Udayapur District, located in the East of Nepal in Asia. The municipality has an elevation of 91 m above sea level and a subtropical climate with annual temperatures ranging from 22 to 40 °C. The mean annual precipitation is approximately 1023 mm (Basin Management Center Koshi, 2022), with the main rainy period occurring from June to September (Rayamajhi et al., 2025). Triyuga covers an area of 547 km^2^ and comprises 16 administrative wards (Fig. 2) or neighborhoods (National Statistics Office, 2022).

**Fig. 1:**
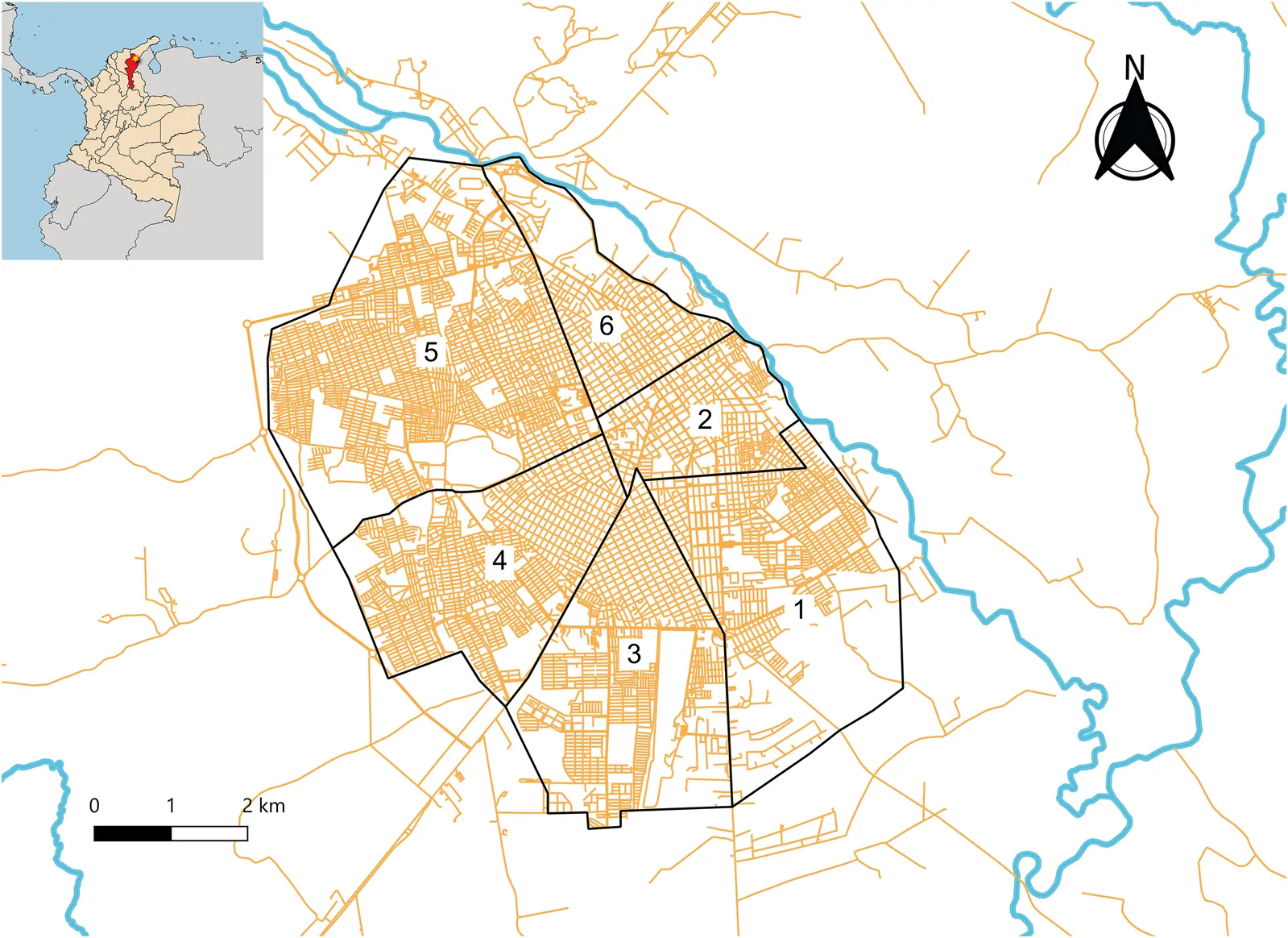
Map of Valledupar, Colombia, showing the six districts (comunas) and the study areas. Base map data from OpenStreetMap. Map created using QGIS 4.0.3. Map lines delineate study areas and do not necessarily depict accepted national boundaries.

**Fig. 2:**
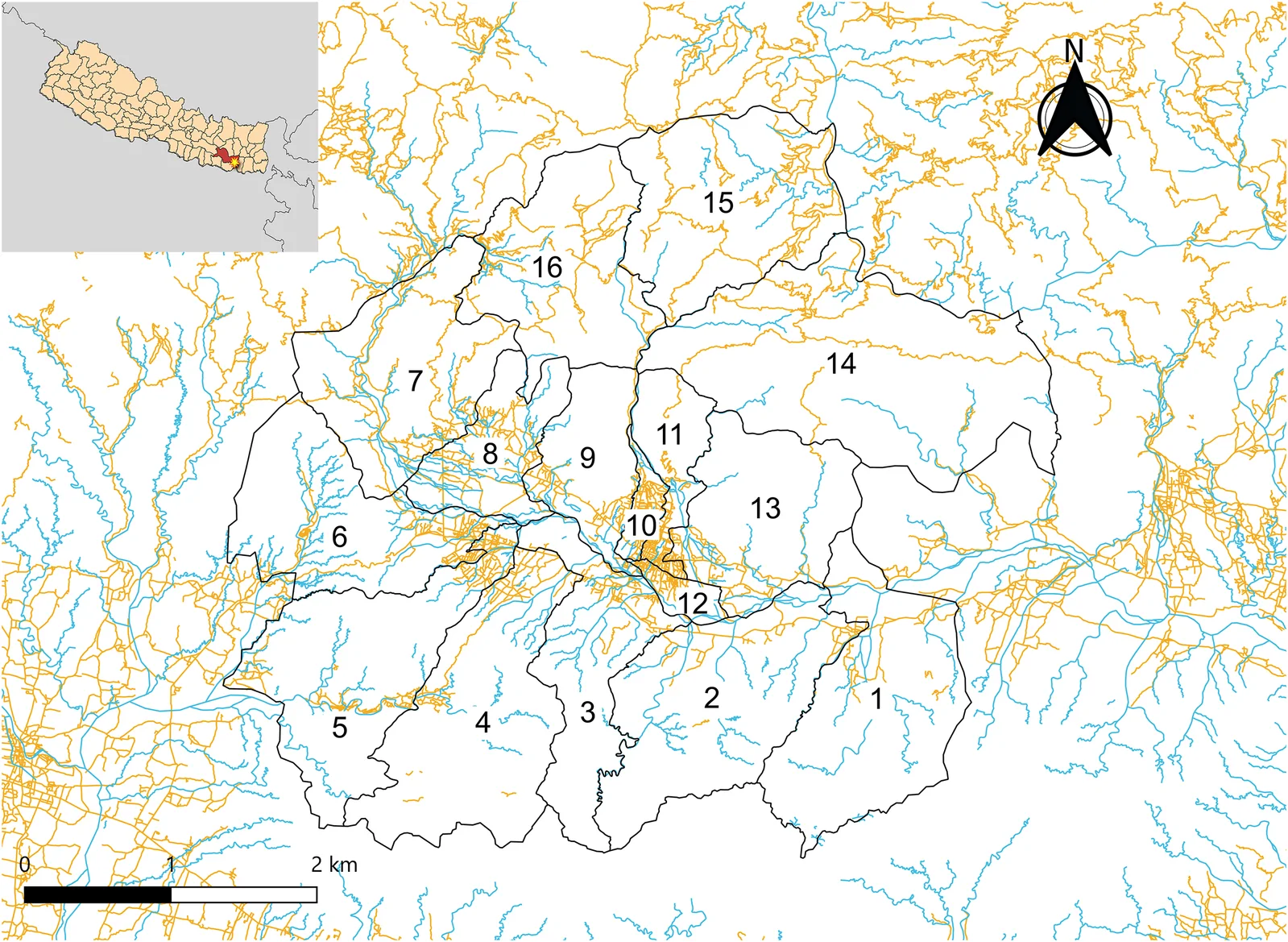
Map of Triyuga, Nepal, showing the 16 wards (clusters) and the study areas. Base map data from OpenStreetMap. Map created using QGIS 4.0.3. Map lines delineate study areas and do not necessarily depict accepted national boundaries.

### 2.2 Sample size

The target population for both studies consisted of households residing in neighborhoods highly infested with *Aedes* spp. larvae. Valledupar is divided into six districts, known as ‘comunas’, each composed of approximately 20 to 50 neighborhoods (clusters), while Triyuga Municipality is divided into 16 wards (clusters). In Valledupar, three districts, highly endemic for dengue, were purposely selected (one neighborhood in Comunas 3, 4, and 5). In Triyuga Municipality, two highly endemic wards were selected (one neighborhood in wards 10 and 12). Within countries, clusters had similar socio-economic conditions, building types, and dengue incidence rates.

The sample size was determined following the WHO recommendations for pupal surveys, which suggest a minimum sample size of 100 houses (Focks and Alexander, 2006). In each cluster (or neighborhood), households were selected randomly using systematic sampling. The sampling interval was calculated by dividing the total number of houses in the neighborhood by the number of expected interviews, resulting in the selection of every 7th house in the three clusters in Colombia and every 28th house in Nepal. This approach reached 300 houses in Valledupar and 121 houses in Triyuga. If a house was closed, the next house to the right was taken.

### 2.3 Study design

The study is predominantly quantitative and cross-sectional, including a pupal/demographic survey (inspection of water containers, household questionnaires) to gather numerical data on mosquito pupae and the demographic characteristics of the population and water containers. Data were collected at a single point in time from the described sample of households.

### 2.4 Data collection

Data were collected using a pupal/demographic survey approach (Focks and Alexander, 2006). The field team included the interviewer, field assistant, and a technician from the local vector control services. In Colombia, the Principal Investigator (PI) was fluent in Spanish; in Nepal, a local resident served as translator. The interviewer was responsible for collecting demographic information through face-to-face interviews with the household head. Another team member inspected all water containers in the intra- and peridomestic area of each house, counting pupae and filling out an entomological questionnaire to obtain data on types of water containers, capacity of the containers and presence of larvae or pupae (Supplementary file 3). An entomological net was used to facilitate pupal counting, particularly in highly infested tanks. For tanks located in poorly lit areas, a flashlight was used to examine the tanks.

Data were collected in July 2024 in Valledupar and in July 2025 in Triyuga. All containers with the presence of larvae or pupae were considered positive for the container and house indices. The number of pupae per container was counted for the pupal indices. In large containers with high pupal density, estimation was done using a calibration factor (Romero-Vivas et al., 2007). The number of pupae encountered was multiplied by the corresponding calibration factor (CF) (according to the water volume of the container) to estimate the number of pupae in large tanks as follows: CF = 2.5 corresponds to 1/3 full; CF = 3.0 corresponds to 2/3 full; CF = 3.5 corresponds to 3/3 full (Manrique-Saide et al., 2011).

### 2.5 Calculation of *Stegomyia* indices

For the interpretation of the *Stegomyia* indices, House Index (HI) and Breteau Index (BI) values > 5%, and Container Index (CI) values > 3% were considered indicative of a high risk of arbovirus transmission (Garjito et al., 2020; Malik et al., 2024). The different *Stegomyia* indices for each surveyed neighborhood were calculated as follows: House Index (HI) = (No. of positive houses for mosquito larvae or pupae/No. of inspected houses) × 100; Container Index (CI) = (No. of positive containers with mosquito larvae or pupae/No. of containers inspected) × 100; Breteau Index (BI) = No. of positive containers with mosquito larvae or pupae/Total no. of houses inspected per 100 houses; Pupae per Person Index (PPI) = Total no. of collected mosquito pupae/Total no. of inhabitants in the inspected houses (Hamid et al., 2021).

## 3 Results

The questionnaire interviews and entomological survey covered 300 households with 506 water storage containers across three clusters in Valledupar (Supplementary file 1) and 121 households with 396 containers in two clusters in Triyuga (Supplementary file 2) (Table 1).

**Table 1:** Entomological survey parameters and indices in Valledupar and Triyuga.

Variable	Valledupar					Triyuga			
	C3	C4	C5	Total	95% CI	C10	C12	Total	95% CI
No. of positive houses	14	20	27	61		12	22	34	
No. of houses	100	100	100	300		60	61	121	
No. of positive containers	18	28	31	77		16	30	46	
No. of containers	146	191	169	506		186	210	396	
No. of pupae	57	33	122	212		136	166	302	
No. of people	349	371	407	1127		311	305	616	
House index (%)	14.00	20.00	27.00	20.33	15.76–24.90	20.00	36.70	28.10	20.10–36.10
Container index (%)	12.33	14.66	18.34	15.21	12.08–18.36	8.60	14.29	11.60	8.40–14.80
Breteau index	18.00	28.00	31.00	25.67	20.73–30.61	26.67	49.18	38.00	27.00–49.00
Pupae per person	0.16	0.09	0.29	0.18	0.07–0.30	0.44	0.54	0.49	0.43–0.54

*Note*: C3, C4, C5, C10, C12 indicate neighborhoods or clusters.

*Abbreviation*: CI, confidence interval.

### 3.1 Water use, control practices, and acceptability

Tap water was the main source of drinking water reported by 96.3% (289/300) of respondents in Valledupar and 81.0% (98/121) in Triyuga (Fig. 3). In Valledupar, some households also reported drinking water directly from ground tanks used to store tap water, a common water-storage practice at this site. In Triyuga, ground tanks were uncommon, and some households stored water in smaller containers. In addition to tap water, 22 households (18.2%) reported using groundwater for drinking. Only 7 households at both sites reported using other sources of drinking water, including water bags, and some households reported more than one source of drinking water.

**Fig. 3:**
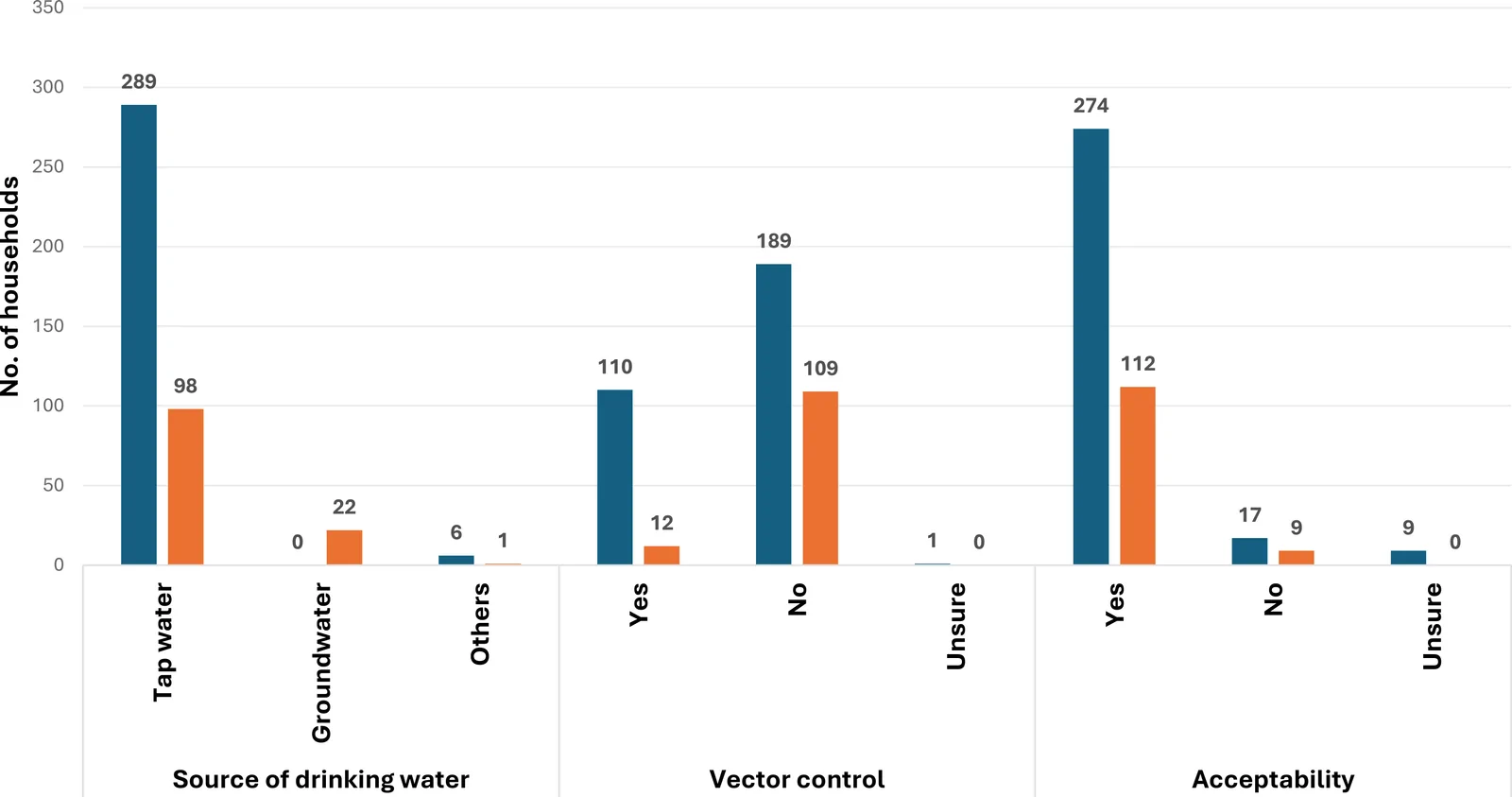
Primary water sources reported by the respondents, household vector control activities, and willingness to accept the implementation of new control measures across both study sites. Absolute household counts are shown in the figure and percentages are reported in the text.

Regarding domestic vector control in water containers, in Valledupar, 63.0% (189/300) of households did not conduct any vector control, but 110 households (36.7%) reported using methods such as bleach or Abate (Temephos) for larval control. In Triyuga, the majority (109 households, 90.1%) reported no vector control. This indicates that water containers in approximately two-thirds of households in Valledupar and the vast majority of households in Triyuga were either unprotected or inadequately maintained.

The household acceptability of vector control methods targeting water containers was also evaluated. A total of 274 households (91.3%) in Valledupar and 112 households (92.6%) in Triyuga expressed willingness to participate, reflecting the interest of the population in new control methods.

The percentages listed above represent the primary water sources reported by the respondents, household vector control activities, and willingness to accept the implementation of new control measures across both study sites. The absolute household counts are shown in Fig. 3.

### 3.2 Entomological indices

The *Stegomyia* indices were calculated using data from all 421 surveyed households (300 in Colombia and 121 in Nepal; Table 1). The HI, CI, and BI values at both study sites exceeded the high-risk thresholds described in *Section* 2.5 (Garjito et al., 2020; Malik et al., 2024).

Valledupar had a mean HI of 20.3%, whereas Triyuga had an even higher mean of 28.1%. The Container Index (CI) had mean values of 15.2% in Valledupar and 11.6% in Triyuga. The Breteau Index (BI) showed the same pattern, with values of 25.67 in Valledupar and 38.0 in Triyuga. The Pupae per Person Index (PPI) was 0.18 in Valledupar and 0.49 (95% CI: 0.43–0.54) in Triyuga, falling within the epidemic transmission risk range of 0.22–2.04 (Jiménez-Alejo et al., 2017) only in Triyuga.

### 3.3 Larval infestation

As shown in Table 2, larval infestation varied across container types and between the two study sites. Larvae were found in small containers such as bottles, buckets, and jars, especially in Nepal.

**Table 2:** Distribution of inspected and larvae-positive water containers by container type in Valledupar and Triyuga.

Container type	Valledupar		Triyuga	
	No. of containers inspected (%)	No. of containers with larvae (%)	No. of containers inspected (%)	No. of containers with larvae (%)
Concrete ground tanks	275 (54.3)	39 (50.6)	2 (0.5)	0 (0)
Plastic ground tanks	58 (11.5)	6 (7.8)	9 (2.3)	1 (2.2)
Metallic ground tanks	3 (0.6)	0 (0)	4 (1.0)	0 (0)
Elevated tanks	1 (0.2)	0 (0)	4 (1.0)	0 (0)
Barrels	0 (0)	0 (0)	47 (11.9)	15 (32.6)
Buckets	96 (19.0)	4 (5.2)	137 (34.6)	6 (13.0)
Cans/jars	6 (1.2)	1 (1.3)	57 (14.4)	4 (8.7)
Flower vases	20 (4.0)	10 (13.0)	17 (4.3)	11 (23.9)
Tyres	1 (0.2)	0 (0)	2 (0.5)	2 (4.3)
Sinks/sewers	2 (0.4)	1 (1.3)	19 (4.8)	1 (2.2)
Bottles	7 (1.4)	4 (5.2)	70 (17.7)	1 (2.2)
Gallon containers	4 (0.8)	1 (1.3)	7 (1.8)	0 (0)
Natural breeding sites	2 (0.4)	2 (2.6)	4 (1.0)	2 (4.3)
Other	31 (6.1)	9 (11.7)	17 (4.3)	3 (6.5)
Total	506 (100)	77 (100)	396 (100)	46 (100)

Concrete ground tanks represented the majority of inspected containers in Valledupar (54.3%) and accounted for more than half of the larvae-positive containers. In Triyuga, the barrels accounted for 32.6% of the larvae-positive containers, while buckets and flower vases, were also very common and contributed to the presence of larvae. However, container abundance did not necessarily correspond to larval infestation. In Valledupar, 4.2% (4/96) of buckets contained larvae compared with 50.0% (10/20) of flower vases, while in Triyuga, 4.4% (6/137) of buckets and 64.7% (11/17) of flower vases were larvae-positive. This suggests that other container characteristics, besides their abundance, may influence their suitability for mosquito breeding.

### 3.4 Pupal infestation

After inspecting all water containers at both sites (506 at Valledupar and 396 at Triyuga), different types of containers were found to yield the greatest number of *Aedes* spp. pupae, although their contributions differed sharply between regions (Table 3). In Valledupar, concrete ground tanks were the most predominant water containers, representing 54.3% of all containers and accounting for 93.4% of all pupae (198/212). A particularly large concrete tank in Cluster 5 of Valledupar contained 112 pupae, fitting in the classification of an REPC (rare but extremely productive container) (Midega et al., 2006). Similarly, in Cluster 12 of Triyuga, one barrel contained 50 pupae, representing another example of an REPC. Reinforcing the association between container size, water capacity, and pupae output, buckets were the second most common container type in Valledupar (19.0%), but they contributed only 2.8% of the total pupae count (6/212). In contrast, ground tanks (concrete, plastic, and metallic) were uncommon in Triyuga (15 containers in total), and only one plastic ground tank contained pupae, accounting for 5 pupae (1.7% of the total).

**Table 3:** Distribution of pupae in the water container types in Valledupar and Triyuga.

Container type	Valledupar				Triyuga			
	No. of containers (%)	No. of containers with larvae	No. of containers with pupae (%)	No. of pupae (%)	No. of containers (%)	No. of containers with larvae	No. of containers with pupae (%)	No. of pupae (%)
Concrete ground tanks	275 (54.3)	39	12 (75.0)	198 (93.4)	2 (0.5)	0	0 (0)	0 (0)
Plastic ground tanks	58 (11.5)	6	2 (12.5)	7 (3.3)	9 (2.3)	1	1 (2.9)	5 (1.7)
Metallic ground tanks	3 (0.6)	0	0 (0)	0 (0)	4 (1.0)	0	0 (0)	0 (0)
Elevated tanks	1 (0.2)	0	0 (0)	0 (0)	4 (1.0)	0	0 (0)	0 (0)
Barrels	0 (0)	0	0 (0)	0 (0)	47 (11.9)	15	11 (32.4)	170 (56.3)
Buckets	96 (19.0)	4	1 (6.2)	6 (2.8)	137 (34.6)	6	3 (8.8)	13 (4.3)
Cans/jars	6 (1.2)	1	0 (0)	0 (0)	57 (14.4)	4	3 (8.8)	6 (2.0)
Flower vases	20 (4.0)	10	0 (0)	0 (0)	17 (4.3)	11	10 (29.4)	51 (16.9)
Tyres	1 (0.2)	0	0 (0)	0 (0)	2 (0.5)	2	0 (0)	0 (0)
Sinks/sewers	2 (0.4)	1	0 (0)	0 (0)	19 (4.8)	1	1 (2.9)	30 (9.9)
Bottles	7 (1.4)	4	0 (0)	0 (0)	70 (17.7)	1	1 (2.9)	1 (0.3)
Gallon containers	4 (0.8)	1	0 (0)	0 (0)	7 (1.8)	0	0 (0)	0 (0)
Natural breeding sites	2 (0.4)	2	1 (6.2)	1 (0.5)	4 (1.0)	2	1 (2.9)	15 (5.0)
Other	31 (6.1)	9	0 (0)	0 (0)	17 (4.3)	3	3 (8.8)	11 (3.6)
Total	506 (100)	77	16 (100)	212 (100)	396 (100)	46	34 (100)	302 (100)

In Triyuga, unlike Valledupar, smaller containers such as barrels, jars, cans, buckets and flower vases were the most productive container types, collectively contributing to 79.5% of all pupae. Buckets were among the most common container types at both sites, representing 19.0% of containers in Valledupar and 34.6% in Triyuga, but contributed minimally to pupal production, accounting for only 2.8% (6/212) and 4.3% (13/302) of pupae, respectively. Small containers such as flower vases, bottles, jars and cans accounted for more pupae in Triyuga (19.2%) than in Valledupar (0.0%). Differences in water storage practices explain much of this variability. In Valledupar, as in many Latin American countries, nearly every household stores water in ground tanks (concrete, plastic, and metallic) for washing and cleaning (Fig. 4), making them the predominant and most productive breeding site. A total of 336 ground tanks were recorded across 300 households, corresponding to an average of 1.12 ground tanks per household, with an estimated average volume of 276 L. In Triyuga, it is more common to store water in smaller containers, particularly buckets (34.6%), followed by bottles (17.7%), cans/jars (14.4%), and barrels (11.9%) (Fig. 5). Together, 184 buckets and barrels were recorded across 121 households, corresponding to an average of 1.5 containers per household.

**Fig. 4:**
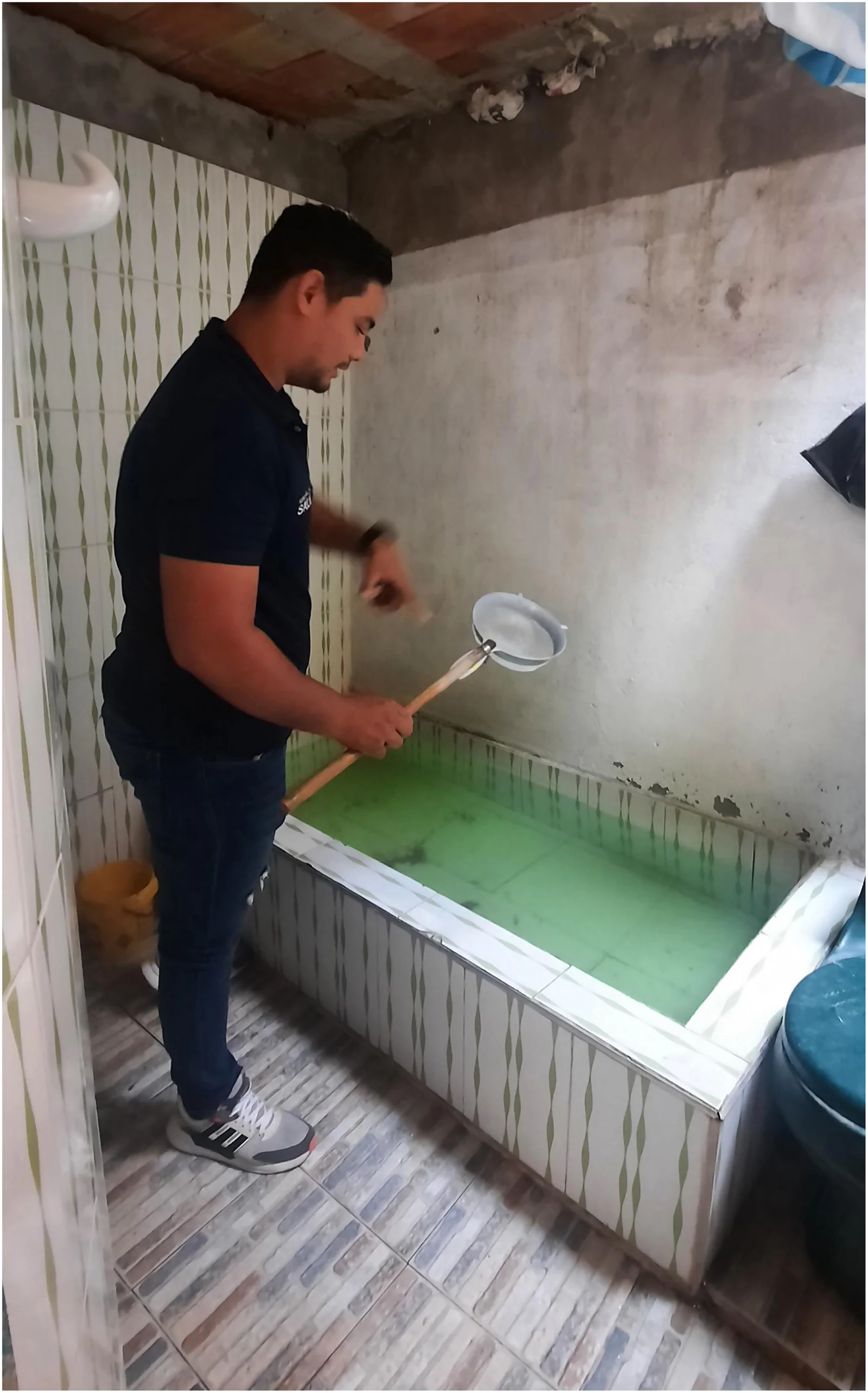
Field collection of *Aedes* spp. pupae in Colombia. Team member collecting pupae from a ground tank in Valledupar.

**Fig. 5:**
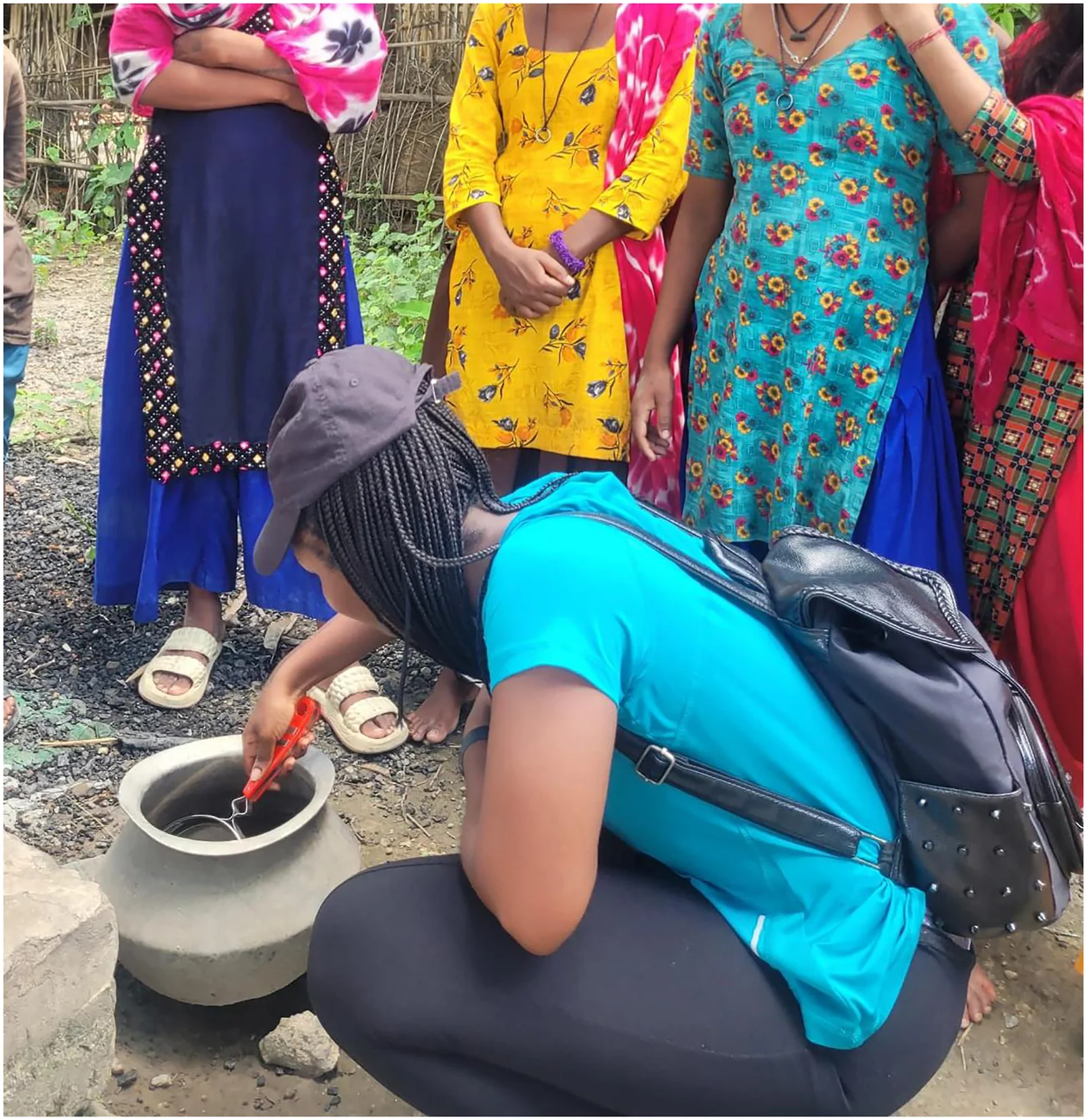
Field collection of *Aedes* spp. pupae in Nepal. Team member collecting pupae from a pot in Triyuga.

### 3.5 Binomial generalized linear model of factors associated with pupal presence

A binomial generalized linear model was conducted to assess factors associated with pupal presence in water containers (Table 4). Pupal presence was the binary outcome, with study site, container type, container location, and container cover included as explanatory variables. Container types were grouped into five categories, excluding several categories with no pupal presence (Table 4).

**Table 4:** Factors associated with pupal presence using a binomial generalized linear model.

Factors	Characteristics				Odds ratio (OR)	
	Level	*N*	*n*	Rate (%)	OR (95% CI)	*P*-value
Study site	Valledupar	506	16	3.16	Reference	
	Triyuga	396	34	8.59	5.85 (2.13–16.06)	<0.001
Container type	Ground tanks^a^	353	15	4.25	Reference	
	Barrels	47	11	23.40	1.46 (0.40–5.28)	0.567
	Buckets	233	4	1.72	0.16 (0.04–0.63)	0.009
	Flower vases	37	10	27.03	3.16 (0.97–10.26)	0.055
	Other	232	10	4.31	0.35 (0.11–1.11)	0.075
Container location	Indoors	270	2	0.74	Reference	
	Outdoors	632	48	7.59	9.14 (2.11–39.64)	0.003
Container cover	Covered	260	5	1.92	Reference	
	Partially covered	28	3	10.71	2.12 (0.41–10.99)	0.372
	Uncovered	614	42	6.84	2.12 (0.76–5.92)	0.151

*Abbreviations*: OR, odds ratio; CI, confidence interval; *N*, number of containers; *n*, number of containers with pupal presence.

a Concrete, plastic, and metallic ground tanks.

The analysis showed that containers in Triyuga had significantly higher odds of pupal presence than those in Valledupar (OR = 5.85, 95% CI: 2.13–16.06, *P* < 0.001). This difference reflects the contrasting distribution of pupae in containers between the two sites. In Valledupar, only 16 of 506 containers contained pupae, which were strongly concentrated in a few container types, whereas in Triyuga, they were found in 34 of 396 containers and across a greater variety of container types. Consequently, pupal presence was too sparse and clustered in Valledupar to assess the different characteristics of containers in a separate model; therefore, data from both sites were analyzed together.

Container location was strongly associated with the presence of pupae. Outdoor containers had significantly higher odds of containing pupae than indoor containers (OR = 9.14, 95% CI: 2.11–39.64, *P* = 0.003). This pattern was consistent at both sites, with all 16 containers containing pupae in Valledupar and 32 of 34 in Triyuga located outdoors, suggesting container location as an important characteristic when identifying potential breeding sites.

Among container types, flower vases showed the highest estimated odds of containing pupae compared with ground tanks, although this association did not reach statistical significance (OR = 3.16, 95% CI: 0.97–10.26, *P* = 0.055). The likelihood of finding pupae in a container did not necessarily correspond to the number of pupae produced. As shown in Table 3, barrels and concrete ground tanks accounted for the largest numbers of pupae in Triyuga and Valledupar, respectively. These results show that the frequency of pupal presence and the number of pupae produced can differ considerably between container types.

Container cover was not significantly associated with pupal presence, as neither partially covered nor uncovered containers showed significant differences compared with covered containers (*P* > 0.05) (Table 4).

## 4 Discussion

This study provides valuable findings about *Aedes* spp. mosquito breeding in two different settings, Valledupar (Colombia) and Triyuga Municipality (Nepal). In low- and middle-income communities, water storage practices vary considerably across regions. The results in Valledupar showed that among all container types, ground tanks were present at an average of 1.12 per house in the surveyed areas, with an estimated average volume of 276 L. In contrast, Triyuga presented a different container profile. Ground tanks were uncommon, and water is more frequently stored in barrels and buckets (Barbazan et al., 2008), accounting for 1.5 containers per house. In Triyuga, small containers such as barrels, flower vases, and sinks/sewers accounted for most pupae, while in Valledupar, ground tanks accounted for more than 90% of all pupae detected. Despite these differences in productive container profiles, the conventional Stegomyia indices indicated substantial infestation at both sites. Valledupar had an HI of 20.3%, CI of 15.2%, and BI of 25.67, while Triyuga had an HI of 28.1%, CI of 11.6%, and BI of 38.0, with all values exceeding the high-risk thresholds used in this study.

However, the PPI showed a different pattern, with Triyuga (0.49) falling within the epidemic transmission risk range of 0.22–2.04, while Valledupar (0.18) remained below it. While HI, CI, and BI measure the frequency of positive houses and containers, without taking into consideration whether they contain very few or many mosquitoes, the PPI measures pupal abundance relative to the human population, providing an indicator more closely related to adult mosquito abundance. At the same time, conventional Stegomyia indices do not identify which container types contribute most to pupal production. Pupal surveys can therefore provide additional information for identifying locally important breeding sites.

The generalized linear model further showed that the odds of pupal presence varied among container types and between study sites, and that higher odds of pupal presence did not necessarily correspond to greater pupal production. This means that vector control services should adapt control measures to the most productive container types in each setting, targeting either large or small water containers.

Another observation was that only 16 of 506 containers in Valledupar and 34 of 396 in Triyuga produced pupae, indicating pronounced clustering, where a disproportionate number of pupae were found in a small number of containers. This clustering was more prominent in Valledupar, and this difference was also observed in the generalized linear model, with higher odds of pupal presence in Triyuga. This pattern reflects previous observations that pupal production tends to concentrate in specific containers rather than being evenly distributed (Barrera et al., 2006). The distribution of pupae was also disproportionate across clusters. Specifically, Cluster 5 in Valledupar had one tank containing 112 pupae, classified as an REPC (rare but extremely productive container), and the same occurred in Cluster 12 in Triyuga, where one barrel contained 50 pupae. This may be due to less frequent water delivery and longer storage periods, allowing containers to remain suitable breeding habitats for longer. As pupae have relatively low mortality and a short development period before adult emergence, the number of pupae can be used as a proxy for adult mosquito production (Focks and Chadee, 1997). The number of adult mosquitoes per person is an epidemiologically relevant indicator of dengue transmission risk (Focks and Chadee, 1997; Manrique-Saide et al., 2011). Therefore, highly productive water containers can contribute to larger adult vector populations, increasing the number of mosquitoes potentially able to feed on human blood and transmit the dengue virus.

The use of drinking-water sources other than direct tap water was relatively uncommon in the surveyed areas. In Valledupar, some households reported drinking tap water stored in ground tanks, reflecting the common practice of storing piped water in these containers, while 6 (2.0%) reported other sources, such as water bags. In Triyuga, where ground tanks were uncommon, 22 households (18.2%) reported groundwater as the source of their drinking water, and one (0.8%) reported another source. Stored water is generally used for washing, bathing, and cleaning. The frequency of water delivery to households may also play an important role; some clusters receive piped water less frequently than others, leading households to store water in more and larger containers, which may increase the availability of suitable mosquito breeding habitats (Knox et al., 2007). Therefore, strategies that focus on preventing mosquito breeding should target the most productive container types in each setting. Containers responsible for *Aedes* spp. breeding are fairly consistent within a country but substantially different between regions, requiring tailored interventions with a prior pupal/demographic survey.

Container location was also an important factor associated with pupal presence. Outdoor containers had substantially higher odds of containing pupae than indoor containers, and almost all containers with pupae at both study sites were located outdoors. This suggests that, in addition to identifying the most productive container types, their location should also be considered when identifying potential *Aedes* spp. breeding sites and planning targeted vector control programmes.

This study confirms that dengue vector control should target the most productive water containers identified through pupal/demographic surveys, taking into account local water storage practices. In Colombia, large containers are common and yield more pupae, whereas in Nepal, smaller containers, including barrels, buckets and flower vases, play a more significant role. The most productive container types can differ between locations, making local identification an important step before implementing vector-control interventions.

The different productive container types identified at the two study sites also suggest different options for targeted interventions. For example, for ground tanks, an intervention using a protective wall coating with pyriproxyfen and alphacypermethrin has been tested in Cúcuta, Colombia, showing promising results for *Aedes* spp. control at a low treatment cost (Cárdenas et al., 2025). For smaller containers, studies from Thailand provide an example similar to the pattern observed in Triyuga, where pupal/demographic surveys identified a limited number of small container types responsible for most *Ae. aegypti* pupal production (Barbazan et al., 2008). Interventions targeting these smaller containers are mostly based on trash elimination and regular cleaning and therefore depend strongly on community awareness and participation. The communities of the two surveyed areas, separated by more than 15,000 km but facing the same problem, were aware of the risks that dengue represents and recognized the need for new control measures. The positive attitude of the population is important, suggesting that they are willing to support the implementation of vector control strategies.

## 5 Conclusions

The findings of this study demonstrate that the most productive *Aedes* spp. breeding sites differ between geographical settings, with large water-storage containers dominating pupal production in Valledupar, Colombia, and smaller containers contributing most in Triyuga, Nepal. These differences highlight the importance of considering local water-storage practices and identifying the container types responsible for the greatest pupal production, rather than assuming that the same breeding sites are equally important across different settings. Targeted vector control strategies should therefore prioritize the specific container types responsible for most pupal production in each context. Pupal/demographic surveys can help identify these productive breeding sites and guide the development of locally adapted interventions. Such context-specific approaches may improve the efficiency of dengue vector control by directing available resources toward the containers contributing most to mosquito production.

## Ethical approval

The research protocol was approved by the Ethical Committee at Albert-Ludwigs-Universität Freiburg (Ref. No. 24-1128-S2) and by local institutions in each participating country (Colombia: Universidad Popular del Cesar, Valledupar; no reference number assigned; Nepal: Nepal Health Research Council, Ref. No. 2358). All respondents signed a written informed consent form prior to participation in the surveys. Informed consent for publication was obtained from all participants. All individuals appearing in photographs included in this manuscript provided explicit consent for the use and publication of their images.

## CRediT authorship contribution statement

**Emerson Barahona:** Conceptualization, Methodology, Investigation, Data curation, Formal analysis, Validation, Visualization, Writing - original draft, Writing - review & editing, Project administration, Resources, Funding acquisition. **Dorinda Nelson-Cofie:** Conceptualization, Methodology, Investigation, Data curation, Formal analysis, Validation, Writing - original draft, Project administration, Resources, Funding acquisition. **Axel Kroeger:** Conceptualization, Methodology, Formal analysis, Validation, Writing - original draft, Writing - review & editing, Supervision, Project administration, Resources. **Kathleen Chavarría:** Investigation, Data curation, Validation, Writing - review & editing, Resources. **Niseth Vence:** Investigation, Validation, Resources. **Megha Raj Banjara:** Investigation, Validation, Writing - review & editing, Supervision, Resources.

## Statement on the use of AI-assisted technologies

During the preparation of this work, the authors used ChatGPT (OpenAI) and Grammarly solely to assist with language editing and grammar correction. After using these tools, the authors reviewed and edited the suggested changes as needed and take full responsibility for the content, interpretation, and conclusions of the manuscript.

## Funding

This work was supported by DAAD PROMOS (University of Freiburg), awarded to Emerson Barahona, and Engagement Global GmbH, awarded to Dorinda Nelson-Kofie. The funders provided support for fieldwork-related travel expenses but had no role in study design, data collection, analysis, interpretation of data, manuscript preparation, or the decision to publish.

## Declaration of competing interests

The authors declare that they have no known competing financial interests or personal relationships that could have appeared to influence the work reported in this paper.

## Data Availability

The data supporting the findings of this study are available in the article and its supplementary files.

## Appendix A Supplementary data

Multimedia component 1 Raw data: Colombia.

Multimedia component 2 Raw data: Nepal.

Multimedia component 3 **Texts S1-S2.** Demographic questionnaire and entomological survey.
