# Supplementary material for: Assessing dengue vector breeding in diverse environments for targeted interventions: Case studies from Colombia and Nepal
Source: Curr Res Parasitol Vector Borne Dis. 2026 Oct 15;10:100449. doi: 10.1016/j.crpvbd.2026.100449 (PMC13717096; doi:10.1016/j.crpvbd.2026.100449)
Supplement: Supplementary file 1 [file mmc3.pdf]

Supplementary file 3

Supplementary Text S1. Demographic questionnaire.

Household questionnaire House ID : _______________

We are working with the health services on a health survey to improve the situation of dengue, chikungunya, and Zika in our city. If you allow us, we would like to ask you a few short questions that will not take much of your time. If you are uncomfortable with any question, you may simply choose not to answer or you may end the interview at any time. We also want to clarify that all information is confidential. Do you agree?

1. Could you provide us with the following details about the people living in this house? (Each person represents one number)

Person	Age	Sex	Years of schooling	Have you had dengue/Zika/chikungunya in the last 12 months?	In which month?	Have you visited any health service for these diseases? Yes/No	Do you study or work far from your home? (sector or travel time in minutes)
1							
2							
3							
4							
5							
6							
7							
8							
9							
10							

Use of water for drinking:

1. Which source does the water you use for drinking or preparing food come from?

Ground tank: ___ Tap Water: ___ Other: ___________________

2. Has the health service carried out any vector control method in the tanks (application of abate (Temephos) or bleach)?

YES: _____ NO: _____ UNSURE: _____

Comment: Dengue, Zika, and chikungunya are transmitted by the same mosquito. You could protect yourself against these mosquitoes using a transparent paint that is non-toxic and approved by the Ministry of Health.

3. Would you like us to paint your tank with a transparent product that protects against mosquitoes, free of charge? It is a new method for controlling dengue, chikungunya, and Zika.

YES: ____ NO: ____ UNSURE: ____

(Spanish version) ID Casa: _____________

Estamos trabajando con La secretaria de Salud Municipal de Valledupar, Universidad Popular del Cesar y Hospital Rosario Pumarejo de López en una encuesta de salud para mejorar la situación del dengue, chikungunya y Zika en nuestra ciudad. Si me permite quisiera hacerle unas preguntas cortas que no le quitarán mucho tiempo. Si usted no se siente bien con alguna de la pregunta, simplemente no conteste o puede terminar la entrevista cuando usted desee. También quiero aclarar que todo es confidencial. ¿Está de acuerdo?

1. ¿Podría darnos los siguientes detalles sobre las personas que viven en la casa? (Cada persona representa un número)

Persona	Edad	Sexo	Años de escolaridad	¿Ha tenido dengue/Zika/chikungunya en los últimos 12 meses?	¿En qué Mes?	¿Ha asistido algún servicio de salud con estas enfermedades? Si/No	¿Estudia o trabaja lejos de su residencia? (sector o distancia en minutos de viaje)
1							
2							
3							
4							
5							
6							
7							
8							
9							
10							

Uso del agua para tomar:

2. ¿De cuál fuente proviene el agua que usa para consumir o preparar alimentos?

Tanque bajo: ___ Agua de la llave: ___ Otros depósitos: ____

3. ¿El servicio de salud ha realizado algún método de control de vectores en los tanques (aplicación de abate o cloro)?

SI: ____ NO: ____ NO SÉ: ____

Comentario: El dengue, Zika y chikungunya son transmitidos por los mismos mosquitos (*Aedes aegypti* y *Aedes albopictus*). Se podría proteger contra estos mosquitos por medio de una pintura transparente que no es tóxica y está aprobada por el Ministerio de Salud.

4 ¿Le gustaría que pintáramos su tanque con un producto transparente que protege contra los mosquitos y de forma gratuita? Es un nuevo método de control de dengue, chikungunya y Zika.

SI: ____ NO: ____ NO SÉ: ____

Supplementary Text S2. Entomological survey.

Day	Month	Year

	House		Workshop
	Health Unit		Store
	Church		Public space
	School		Other

Neighbor hood: ____________________ **Address:** ____________________ **Survey number:** __________

Inspector: ______________________________

		Water tanks					Other water containers										Details of each water tank					Entomological indices		Measures			
		Ground tanks																									
		**Plastic**	**Concrete**	**Metalic tanks**	**Elevated Tank**	**Used for drinking**	**Barrels**	**Buckets**	**Cans/Jars**	**Flower Vases**	**Tyres**	**Sink/Sewer**	**Bottles**	**Gallon containers**	**Natural breeding sites**	**Others:**	**Water source:** (1=Tap, 2=Rain, 3=Other)	**Location of the container** (1=Inside, 2=Outside)	**Shading** (1=Yes, 2=Partially, 3=No)	**Covered** (1=Yes, 2=Partially, 3=No)	**Level of water** low1/3; Medium2/3; full3/3	**Larvae** Positive=(+),negative=(-)	**Pupae count** Total number	**Capacity (Liters)**	**Length**	**Width**	**Height**
**One container in each row**	**1**																										
	**2**																										
	**3**																										
	**4**																										
	**5**																										
	**6**																										
	**7**																										
	**8**																										
	**9**																										
	**10**																										
	**11**																										
	**12**																										
	**13**																										
	**14**																										
	**15**																										
